# NHS-POx-loaded patch versus fibrin sealant patch in a porcine robotic liver bleeding model

**DOI:** 10.1186/s12893-023-02159-4

**Published:** 2023-08-28

**Authors:** Mathieu D’Hondt, Edwin A. Roozen, Frederiek Nuytens, Johan Bender, Alexandre Mottrie, Kevin Bauwens, Stuart J. Head

**Affiliations:** 1 Department of Digestive and Hepatobiliary/Pancreatic Surgery, AZ Groeninge Hospital, President Kennedylaan 4, 8500 Kortrijk, Belgium; 2 GATT Technologies, Nijmegen, The Netherlands; 3 ORSI Academy, Ghent (Melle), Belgium

**Keywords:** Bleeding, Hemostasis, NHS-POx patch, GATT-Patch, TachoSil, Fibrin sealant patch, Liver surgery, Robotic

## Abstract

**Background:**

The management of bleeding is paramount to any surgical procedure. With the increased use of less invasive laparoscopic and robotic methods, achieving hemostasis can be challenging since the surgeons cannot manually apply hemostatic agents directly onto bleeding tissue. In this study, we assessed the use of a pliable hemostatic sealant patch comprising fibrous gelatin carrier impregnated with poly(2-oxazoline) (NHS-POx) for hemostasis in robotic liver resection in a porcine bleeding model.

**Methods:**

The NHS-POx-loaded patch (GATT-Patch), was first evaluated in a Feasibility Study to treat surgical bleeding in 10 lesions, followed by a Comparative Study in which the NHS-POx patch was compared to a standard-of-care fibrin sealant patch (TachoSil), in 36 lesions (superficial, resection, or deep injuries mimicking metastasectomies). For each lesion type, the NHS-POx and fibrin sealant patches were used in an alternating fashion with 18 lesions treated with NHS-POx and 18 with the fibrin patch. Animal preparation and surgical procedures were consistent across studies. The primary outcome was time to hemostasis (TTH) within 3 min for the Feasibility Study and within 5 min for the Comparative Study.

**Results:**

In the Feasibility Study, 8 of the 10 NHS-POx-treated lesions achieved hemostasis at 30 s and 3 min. In the Comparative Study, all 18 NHS-POx patch-treated lesions and 9 of the 18 fibrin sealant patch-treated lesions achieved hemostasis at 5 min. Median TTH with NHS-POx vs fibrin sealant patch was 30 vs 300 s (*P* < 0.001).

**Conclusions:**

In this animal study, hemostasis during robotic liver surgery was achieved faster and more often with the NHS-POx loaded vs fibrin sealant patch.

**Supplementary Information:**

The online version contains supplementary material available at 10.1186/s12893-023-02159-4.

## Background

For many surgical indications open procedures are being replaced by less invasive laparoscopic and robotic methods. Minimally invasive liver surgery is internationally accepted as a safe and feasible alternative to open hepatectomy. It is associated with lower perioperative morbidity, less blood loss and shorter hospitalization time, while maintaining equivalent oncological outcomes [1–4].

During any type of surgery there may be surgical bleeding for which hemostasis is difficult to achieve, necessitating the use of topical hemostatic agents [5–10]. However, the risk of substantial bleeding during minimally invasive liver surgery is a major concern. The magnitude of intraoperative blood loss is independently associated with worse perioperative and long-term outcomes [11–13]. Control of bleeding with topical hemostatic agents may be particularly challenging in these minimally invasive procedures since surgeons cannot manually apply hemostatic agents directly to the bleeding site. This could result in prolonged bleeding, requirement for blood transfusions, and potentially conversion from minimally invasive to open surgery.

In order for a topical hemostatic agent to be effective in minimally invasive surgery, the agent should be flexible and easy to handle for introduction through a trocar, successful navigation towards a bleeding site with subsequent adequate positioning, and be able to handle at least moderate bleeding severity to prevent the need for conversion from minimally invasive to open surgery. However, currently available topical hemostatic agents have a number of limitations, such as not being approved for minimally invasive use, not user-friendly to achieve successful introduction, navigation, and positioning, and not sufficiently effective for moderate or severe bleedings [14–16]. During minimally invasive surgeries, hemostatic powders or flowable matrix-based absorbable hemostats are often preferred, but these generally lack the ability to stop problematic bleedings [17].

GATT-Patch (GATT Technologies BV, Nijmegen, The Netherlands) is a new poly(2-oxazoline) (NHS-POx)-based hemostatic sealant patch that has specific design features for hemostatic performance even in severe bleeding and with flexibility and pliability to allow ease of use in minimally invasive procedures [18, 19]. In previous animal studies, use of this NHS-POx-loaded patch has demonstrated safety and efficacy during open procedures on the liver and spleen, and during robotic procedures on the kidney [19, 20].

In order to evaluate the efficacy of the NHS-POx loaded patch in minimally invasive surgery, we performed robotic liver resection in a porcine bleeding model compared to the standard-of-care fibrin sealant patch (TachoSil), for the achievement of hemostasis.

## Methods

### Study designs

Initial use of the NHS-POx-loaded patch was evaluated in a Feasibility Study in a porcine model with 2 animals. The primary objective of the Feasibility Study was to familiarize a single surgeon experienced with robotic procedures with NHS-POx patches, who regularly uses the fibrin sealant patch, i.e. control arm, during robotic liver surgery in clinical practice. A specific surgical procedure was not prescribed for lesion creation and location of bleeding sites, so that the surgeon would be trained on the NHS-POx patch in a variety of scenarios without limitations.

Subsequent to the Feasibility Study, the NHS-POx-loaded patch was compared to the fibrin sealant patch in 4 animals in which 36 lesions were attempted (Comparative Study), which included specific procedures for lesion creation and location of bleeding sites. For each lesion type, the NHS-POx and fibrin sealant patches were used in an alternating fashion with 18 lesions treated with NHS-POx and 18 with the fibrin patch. No a priori sample size calculation was performed.

The same methods were used in both studies for the preparation and treatment of the animals. The studies were approved by the Ethics Committee and performed in the ORSI facility at Proefhoevestraat 18, 9090 Melle, Belgium. All ORSI animal programs are in compliance with Belgian and European legislation on protection of animals used for scientific purposes.

### Study devices

We examined the efficacy of an NHS-POx-loaded patch to achieve hemostasis in minimally invasive robotic porcine liver bleeding model. For comparison, a fibrin sealant patch (TachoSil Patch(Corza Medical, Westwood, Massachusetts, USA) was used, which is approved in the European Union for minimally invasive use as a method to achieve hemostasis during surgical procedures [14].

### Study animals

Six (5 male, 1 female) Rattlerow Seghers (Suffolk, UK) pigs, age 13–15 weeks and weighing 50–60 kg, were used during these terminal experiments. The animals were sedated using ketamine (10 mg/kg), medetomidine (40 µg/kg) and morphine (0.1 mg/kg), and subsequently anesthetized with propofol (1–3 mg/kg). Intubation was performed after 10% lidocaine spray application on the trachea. Anesthesia was maintained during the procedure using isoflurane/sevoflurane in 55% oxygen. Intraoperatively the animals further received Ringer’s lactate solution (10 ml/kg/h). A mean arterial blood pressure higher than 60 mm Hg was maintained using L-noradrenaline, if required. Animals were monitored by and for electrocardiography, pulse oximetry, capnography and spirometry, invasive blood pressure, and temperature. At the end of the experiment, animals were euthanized with a lethal dose of pentobarbital (50 mg/kg) or T61 (1 ml/10 kg).

### Procedures

Animals were placed in supine position on the operating table in front of a Da Vinci Robot (Intuitive, Sunnyvale, California, USA). After anaesthesia, using a Veress needle and CO2 influx, 3 standard robotic 8-mm ports were introduced across the abdomen for the robotic arms, 1 standard 12-mm port for the camera, and 1 (in the Comparative Study) or 2 (in the Feasibility Study) standard 12-mm ports were placed for suction, introduction of sutures if required, and introduction of gauzes and hemostatic patches (NHS-POx loaded or fibrin sealant). The intra-abdominal pressure was set at 10 mm Hg in the Feasibility Study and 8 mm Hg in the Comparative Study during the entire procedure.

**Fig. 1:**
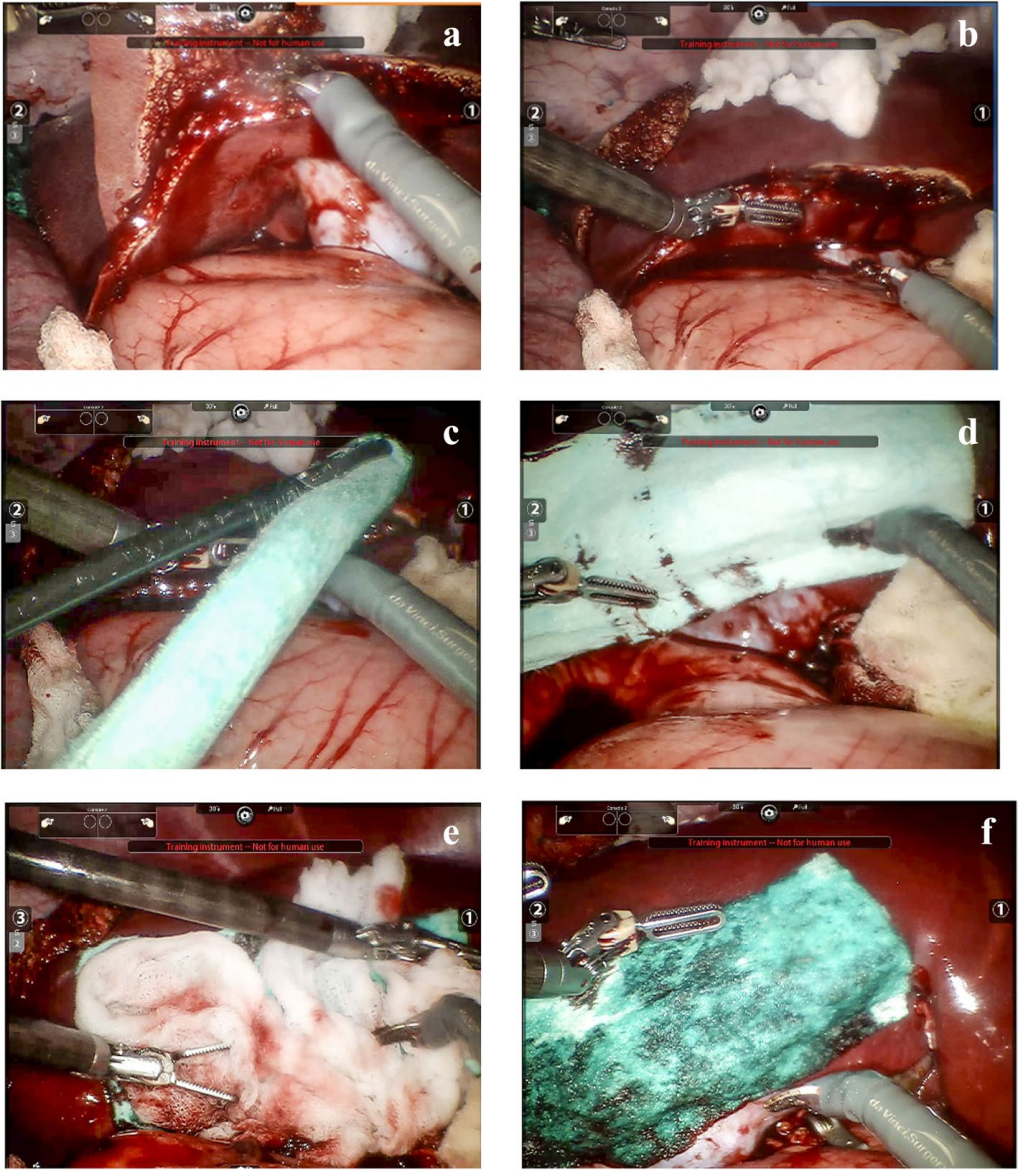
Creation and treatment of liver resection bleeding lesion. **a**. Creation of the lesion; **b**. bleeding severity; **c**. introduction of NHS-POx patch; **d**. application of NHS-POx patch; **e**. pressure with wet gauze; **f**. hemostasis achieved with NHS-POx patch. Fenestrated bipolar forceps and monopolar curved scissors were used in addition to a training instrument not for human use

Sharp non-cauterizing scissors, sharp monopolar-cauterizing scissors, or bipolar vessel sealing systems were used to create lesions of the liver parenchyma, and the main vessels of the liver in the Feasibility Study (Fig. 1). The goal was to create 4–6 lesions per animal in the Feasibility Study and 8 10 lesions per animal in the Comparative Study, depending on the size, anatomy and position of the liver, the location of the lesions, and the hemodynamic status of the animal.

After lesion creation, the severity of bleeding was adjudicated according to the Severity Bleeding Surface Scale (SBSS) by consensus of 2 trained investigators (E.A.R. and S.J.H.) that hold a certificate on the adjudication of bleeding on this scale of 0–5 bleeding severity: 0 represents no bleeding, 1 represents minimal bleeding, 2 represents mild bleeding, 3 represents moderate bleeding, 4 represents severe bleeding, and 5 represent extreme, life-threatening bleeding [21]. Hemostasis was considered to be an SBSS of 0.

NHS-POx-loaded and fibrin sealant patches were cut to the appropriate size so that it overlapped non-bleeding tissue by at least 1 cm on all sides. The NHS-POx patch was subsequently rolled over its long axis and grasped at the most distal tip to be introduced through a 12-mm size trocar. After navigation to the bleeding and unrolling, the NHS-POx patch was positioned on the bleeding site and pressure applied for 30 s with a saline-wetted gauze per Instructions for Use (IFU), after which hemostasis was checked by carefully removing the gauze (Fig. 1**)**. If hemostasis was not yet achieved, either additional pressure, or additional (pieces of) NHS-POx patch was applied. Hemostasis was checked at predetermined times of 30 s, 1 min, and 3 min in both the Feasibility and Comparative Study, with the additional primary endpoint of 5 min only for the Comparative Study. The time to hemostasis (TTH) was considered to be the time that hemostasis was achieved and maintained until the end of the procedure, which was up to about 1 three-fourths hours after creation of the first lesion.

In the Feasibility Study, the NHS-POx-loaded patch was primarily used to train the surgeon, but the fibrin sealant patch was used as a control on 1 lesion for direct comparison of ease of use in a single surgery. For this lesion, the fibrin sealant patch was folded and placed inside a cut-off finger from a surgical glove, to improve ease of introduction through the trocar, after which it was grasped at the most distal tip and introduced through a 12- mm trocar. In the Comparative Study, the fibrin sealant patch was folded in a zigzag shape over its long axis and introduced directly without placing it in the surgical glove. After introduction, the patch was navigated to, and positioned on the bleeding site and pressure was applied for 3 min (per IFU) with a saline-wetted gauze, after which the gauze was carefully removed and hemostasis was checked. If hemostasis was not yet achieved, either additional pressure, or additional (pieces of) fibrin sealant patch was applied up to the time of the primary endpoint of 5 min when hemostasis was always checked.

### Outcomes

The primary endpoint for both studies was hemostatic performance as determined by the TTH, with a pass or fail at 3 min in the Feasibility Study and at 5 min for the Comparative Study. Since the fibrin sealant patch has a prescribed 3-min application time when hemostasis is checked, we extended the TTH outcome to 5 min in the Comparative Study to confirm complete hemostasis was achieved with no rebleeding. Additional secondary outcomes included the use of the NHS-POx-loaded patch and fibrin sealant patch with a measure of pass (successful procedure without damaging the patch) or fail (patch could no longer be used). These measures included preparation and introduction of the patch through a trocar, navigation to the bleeding site, unrolling or unfolding of the patch, positioning on the bleeding site, application of the patch with a wet gauze and the removal of the gauze after placement.

### Statistical analyses

Results are provided as descriptives. The difference in median TTH between groups was calculated with the Mann–Whitney U test, with a *P* value of < 0.05 defining statistical significance. When hemostasis was not achieved and a TTH was not available, a time of 305 s (5 s longer than the primary endpoint of hemostasis at 5 min) was used for input in the calculation.

## Results

### Feasibility study

A total of 8 liver lesions were created (4 resections, 4 mimicking metastasectomies). The NHS-POx-loaded patch was used in 7 lesions and the fibrin sealant patch was attempted in 1 lesion. The median interquartile range (IQR) SBSS was 4 (3–4). The NHS-POx patch achieved hemostasis in 30 s in 6 of 7 (86%) lesions (Table 1). One lesion, with extreme, life-threatening bleeding (SBSS 5), required an additional NHS-POx patch placement after a piece of patch came loose and hemostasis was achieved at 8 min and did not meet the primary endpoint.

**Table 1:** Feasibility study lesion details

Lesion	Lesion characteristics and size	Patch	Patch Size	SBSS	TTH (s)	Remarks
1	Metastasectomy 1 × 1 cm and 0.8 cm depth	NHS-POx	1 × 2 cm in depth of the lesion and 4 × 5 cm on top	3	30	None
2	Resection 5 × 1.5 cm and 2 cm depth	NHS-POx	6 × 5 cm	4	30	None
3	Resection 6 × 2 cm and 3 cm depth	NHS-POx	1 full patch (10 × 5 cm)	3	30	None
4	Metastasectomy 3.5 by 3 cm and 2 cm depth	NHS-POx	1 full patch (10 × 5 cm)	2	30	Piece of patch came loose upon removal of gauze, but hemostasis achieved
5	Metastasectomy 2.5 by 2.5 cm and 2 cm depth	NHS-POx	1 full patch (10 × 5 cm) in depth of the lesion and 5 × 5 cm on top	4	30	None
6	Portal vein – 0.25 × 0.25 cm	NHS-POx	1/3 patch	4	30	None
7	Metastasectomy 1.5 by 1.5 cm and 1.5 cm depth	NHS-POx	3.5 × 5 cm in depth of the lesion and 3.5 × 5 cm on top	4	30	None
8	Resection 3 by 2 cm and 3 cm depth	NHS-POx	6.5 × 5 cm and 5 × 5 cm	5	480	Piece of patch came loose, additional patch applied resulting in hemostasis
9	Resection 3 by 2 cm and 3 cm depth	Fibrin sealant	4.8 × 8 cm and 4.8 × 4.8 cm	4	Hemostasis not achieved	First patch broke in trocar, no hemostasis achieved
9	Resection 3 by 2 cm and 3 cm depth	NHS-POx	1 full patch (10 × 5 cm)	4	30	Used after TachoSil failed
10	Vena cava	NHS-POx	NA	5	Hemostasis not achieved	First attempt failed; second attempt achieved hemostasis. After removal of gauzes and manipulation of bleeding site, rebleeding occurred and hemostasis was not achieved with 2 additional attempts

*NA* not available, *SBSS* Severity Bleeding Surface Scale, *TTH* time to hemostasis

For the 1 lesion treated with the fibrin sealant patch, it was introduced through the trocar before creating the bleeding lesion. During introduction, a small part of the patch broke off and remained in the trocar. After the lesion was created (SBSS 4), and as the fibrin sealant patch was removed from the glove, additional pieces broke off, but could still be placed on the bleeding site. After 2 attempts of holding pressure with a wet gauze for 3 min, hemostasis was achieved, not meeting the primary endpoint. Subsequently, breakthrough bleeding occurred and eventually the fibrin sealant patch was removed and a NHS-POx-loaded patch was placed with hemostasis achieved in 30 s.

After the liver resection procedures, 1 vascular lesion was created in each animal in a large vein (portal vein and inferior vena cava) to attempt to treat severe and extreme, life-threatening bleeding. The cut in the portal vein (SBSS 4) was successfully treated with the NHS-POx-loaded patch with hemostasis achieved at 30 s. The cut in the vena cava (SBSS 5) was large (about 5 mm) and could only be visualized with several robotic arms, which required removing tools that would otherwise be used to perform suction, grasp gauzes, or introduce the patch. Hemostasis was initially achieved, but after removal of gauzes with manipulation of the site, rebleeding occurred. With compromised visualization and after multiple attempts, persistent hemostasis could not be achieved.

### Comparative study

**Fig. 2:**
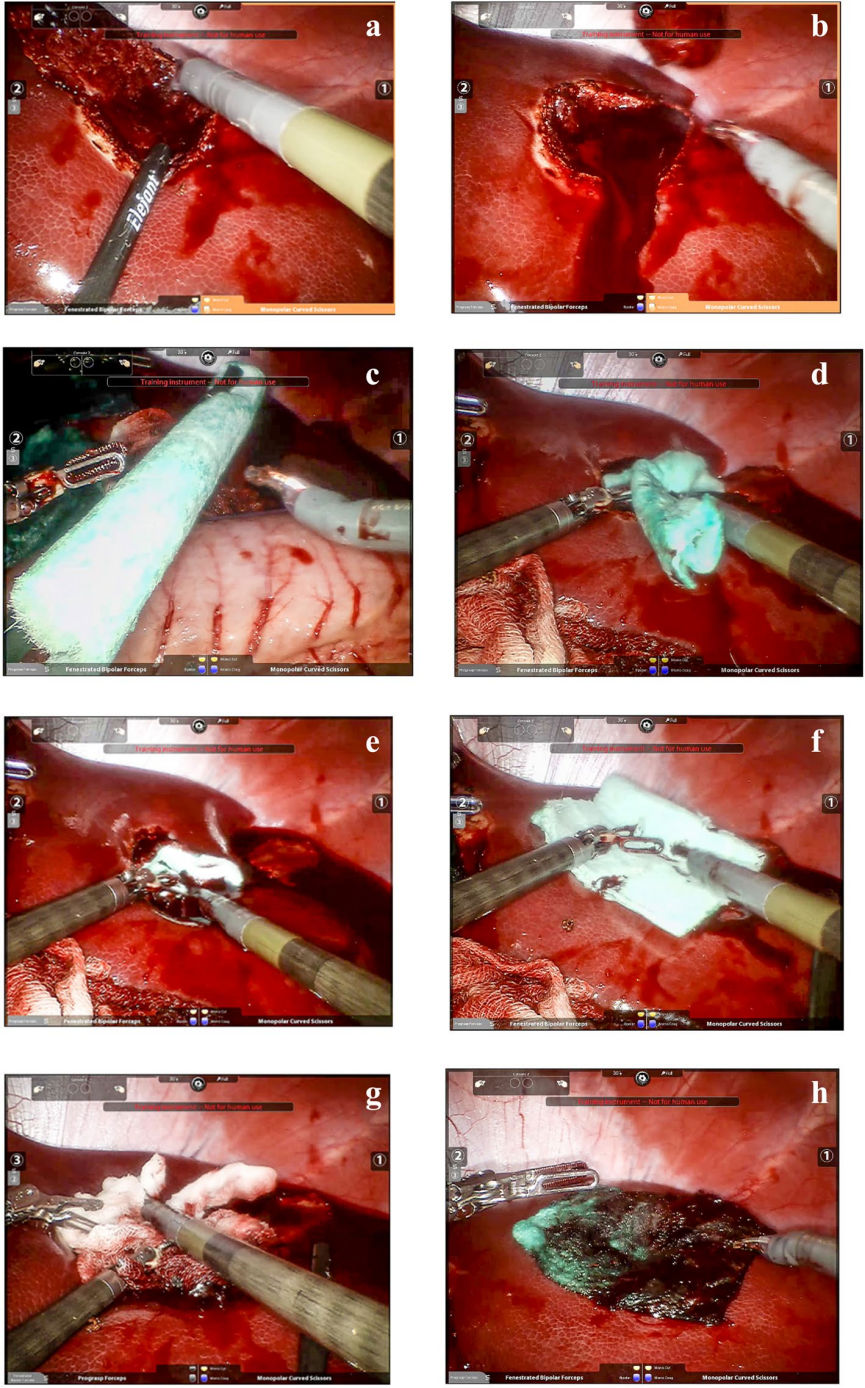
Creation and treatment of liver metastasectomy bleeding lesion. **a**: creation of the lesion; **b**: bleeding severity; **c**: introduction of NHS-POx patch **d**, **e**: application of first piece of NHS-POx patch in depth of lesion; **f** application of second piece NHS-POx patch on top of first one; **g**: pressure with wet gauze; **h**: hemostasis achieved with NHS-POx patch. Fenestrated bipolar forceps and monopolar curved scissors were used in addition to a training instrument not for human use

A total of 36 lesions were created and treated with NHS-POx-loaded patch (*n* = 18) or fibrin sealant patch (*n* = 18). Of these, 16 were superficial lesions (*n* = 8 for both arms), 8 (*n* = 4 for both arms) were partial liver resections (see Fig. 1 for example), and 12 (*n* = 6 for both arms) were deep mimicking metastasectomies (see Fig. 2 for example). For the superficial lesions, the median SBSS was 2 in each group, for the resections, the median SBSS was 3 in each group, and for mimicking the deep metastasectomies, the median SBSS was 4 in each group (Table 2). The median (interquartile range [IQR]) SBSS for all lesions was 3 (2–4) for both groups.

**Table 2:** Comparative study lesion details

	NHS-POx Patch *N* = 18		Fibrin Sealant Patch *N* = 18	
	SBSS	Time to hemostasis (s)	SBSS	Time to hemostasis (s)
Superficial	2	30	2	Not achieved
	3	150	3	Not achieved
	2	30	2	180
	2	30	2	180
	2	30	2	180
	1	30	2	300
	2	30	1	180
	2	30	2	Not achieved
Resection	3	280	3	Not achieved
	2	30	2	Not achieved
	3	30	4	Not achieved
	4	30	3	180
Deep metastasectomy	4	280	3	Not achieved
	5	30	5	180
	4	30	5	300
	4	30	4	Not achieved
	5	30	4	300
	3	30	3	Not achieved
All lesions, median (IQR)	3 (2–4)	30 (30–30)	3 (2–4)	300 (180 – not achieved)

*IQR* interquartile range, *SBSS* Severity Bleeding Surface Scale

**Fig. 3:**
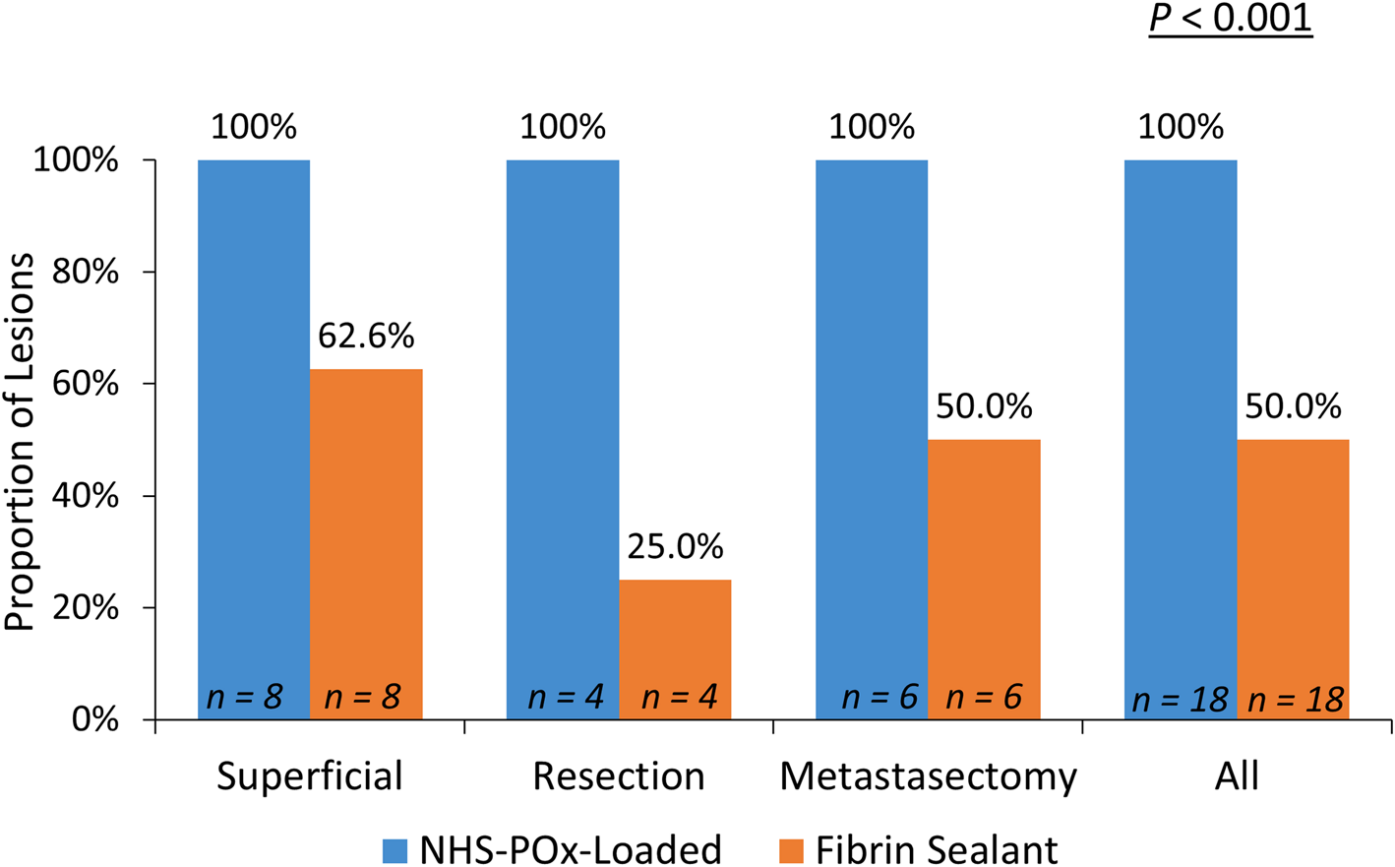
Proportion of treated lesions achieving hemostasis within 5 minutes. The proportion of treated superficial, resection and deep metastasectomy lesions that met the primary objective of achieving hemostasis within 5 min. *P* value calculated using Mann–Whitney U test

Hemostasis was achieved at the primary endpoint at 5 min in all 18 (100%) NHS-POx-loaded patch lesions and 9 of the 18 (50%) of the fibrin sealant patch lesions (Fig. 3). NHS-POx patch achieved hemostasis in 15 of 18 lesions (83%) after the IFU-indicated 30 s pressure and the fibrin sealant patch achieved hemostasis in 6 of 18 (33%) lesions after the IFU-indicated 3 min of pressure. The overall median (IQR) TTH was 30 s (30 s – 30 s) in the NHS-POx patch treated lesions and 300 s (180 s – not achieved) for the fibrin sealant lesions, with a *P* value of < 0.001 (Table 2). The mean TTH was shorter with NHS-POx patch for all lesion types.

### Usability

For all 28 liver and vascular lesions treated with the NHS-POx-loaded patch in both studies, 42 pieces of or full-sized NHS-POx patch were introduced. The introduction through the trocar was successful without issues in all but 2 cases: (1) when used not in accordance with IFU, there was a small tear of a piece of NHS-POx patch that was folded over its short axis instead of rolled over its long axis (per IFU) for introduction, making it thicker, though it could still be used for successful hemostasis; (2) NHS-POx patch was temporarily adhering within the trocar due to excessive wetness of the trocar, but could be delivered and used successfully for hemostasis with a minor delay of less than 30 s. Subsequent navigation to the lesion, unfolding the patch and positioning on the bleeding site was successful without adhesion of the patch to other tissue, tearing, or difficulty to position.

For all 19 lesions attempted with the fibrin sealant patch, and used in accordance with IFU, the patch could be introduced through the trocar, and it was possible to navigate to the lesion and unfold the patch and position on bleeding site. Yet in 6 cases, the surgeon reported more difficulty to unfold the fibrin sealant patch as compared to NHS-POx patch. In all 19 cases, it was possible to position the patch on the bleeding site and loosen the gauze from the patch, although with removal of the gauze the patch frequently loosened from the tissue at the edges.

## Discussion

There is an unmet need of surgical tools to treat bleeding during minimally invasive liver surgery to further optimize clinical outcomes [11–13]. In these first studies of an NHS-POx-loaded patch in minimally invasive robotic liver resections in a porcine bleeding model, hemostasis of liver bleeding, including severe bleeding, can be achieved quickly and reliably. It could be introduced through the 12-mm trocar in all lesions, could be easily navigated to and be positioned onto the bleeding site with adequate pressure upon application. When compared to a standard-of-care hemostatic patch, hemostasis at 5 min was achieved in 25 of the 26 (96%) NHS-POx patch lesions as compared to 9 out of 19 (47%) fibrin sealant patch lesions. Moreover, the median TTH was significantly shorter with the NHS-POx patch vs the fibrin sealant patch (30 vs 300 s; *P* < 0.001).

Several important differences exist between the NHS-POx-loaded patch and the fibrin sealant patch. First, the fibrin sealant patch is a biologically active patch with thrombin and fibrinogen to induce blood clotting, while the NHS-POx patch does not include any biologically active components; it consists of a synthetic polymer impregnated in a gelatin carrier, which allows the patch to adhere strongly to the bleeding tissue and provides a hemostatic seal at the site of application. Second, the carrier for the fibrin sealant patch is an equine collagen, which is brittle and not easily handled during minimally invasive procedures in this study. In fact, in 3 lesions, pieces of the active components of the fibrin sealant patch broke off in the trocar or while placing the patch. On the contrary, the carrier of the NHS-POx patch is a fibrous structure of porcine gelatin which makes it flexible and pliable. In our study, all NHS-POx and fibrin sealant patches could be delivered through the trocar and navigated to the lesion and positioned on the bleeding site. Despite the surgeon being experienced with the fibrin sealant patch, it was noted that the patch was difficult to deliver in some lesions and would loosen at the edges with removal of the gauze. Third, the NHS-POx patch does not require additional preparation after rolling to introduce into the trocar, compared with careful folding and sometimes introduction in a cut-off finger from a surgical glove for the fibrin sealant patch.

These design aspects result in a number of clinical differences, including the time required by the surgeon to apply pressure after placement. The NHS-POx-loaded patch has an application period of 30 s to achieve hemostasis, versus 3 min for the fibrin sealant patch, and in our studies, 26 NHS-POx patch liver applications were performed with 100% success (85% achieved hemostasis in 30 s). While in the lesions treated with the fibrin sealant patch, only 30% achieved hemostasis at 3 min (per IFU), and only 50% reached hemostasis at the primary endpoint of 5 min.

The NHS-POx-loaded patch achieved TTH of 30 s consistently across all 3 lesion types (Table 2), while the fibrin sealant patch often required additional pressure holding if hemostasis was not achieved. This difference in TTH, especially for the resection in a thin porcine liver, demonstrates the flexibility and ease-of-use of the NHS-POx-loaded patch to fold around the bleeding lesion and position on complex bleeding in minimally invasive procedures. In deep injuries mimicking metastasectomies, with the NHS-POx patch, 5 of the 6 achieved a TTH of 30 s, and 1 of the 6 achieved a TTH of 3 min with the fibrin sealant patch. In deep lesions, the fibrous NHS-POx patch can be placed into the depth of the lesion to "plug" the hole, and additional pieces can be added, if necessary. This is not possible with the brittle traditional flat surfaced fibrin sealant patch, thus is a unique feature of the NHS-POx patch that provides an advantage even over flowable matrix-based absorbable hemostats that are often used in these types of bleeding [22].

In this study we demonstrated that the NHS-POx-loaded patch may not be limited to mild to moderate bleeding like other hemostatic agents, but can also be successfully used in severe bleeding. Severe bleeding remains a challenge in liver surgery, especially using a minimally invasive laparoscopic approach [23]. Few products are currently available in clinical practice and are not as effective in severe bleeding. In our studies, there were 17 severe or extreme, life-threatening bleeding (SBSS 4–5) liver resection and lesions mimicking deep metastasectomies (11 in the NHS-POx patch group [including the rescue] and 6 in the fibrin sealant patch group). One bleeding was first attempted with the fibrin sealant patch, but required rescue treatment with the NHS-POx patch. Of the 11 NHS-POx patch lesions, 9 (82%) achieved hemostasis in 30 s; of the 6 fibrin sealant patch lesions, 1 achieved hemostasis in 3 min. Our studies included 2 vascular lesions for which the use of the NHS-POx patch was considered experimental in nature for potential future studies. Nevertheless, in the portal vein lesion with SBSS 4, the NHS-POx patch achieved hemostasis in 30 s. In the vena cava lesion with SBSS 5, the NHS-POx patch could not be placed accurately on the bleeding site due to the difficulty in visualizing the bleeding site. The use of the NHS-POx patch on vascular lesions was not the main objective of this study, however, future studies are warranted as a primary method for achieving hemostasis or as an adjunct in combination with suturing.

Our study was performed in a porcine model for the initial use of a new hemostatic patch during minimally invasive robotic liver surgery. As the NHS-POx patch does not currently have regulatory approval for clinical use (i.e., conformité européenne [CE] Mark or United States Food and Drug Administration [FDA] approval), an evaluation of minimally invasive use was limited to an animal model. For the evaluation of a hemostatic agent, continuous blood flow is required and although ex-vivo perfusion of selective organs provides an alternative to live animal surgery, the translational value is limited, specifically in the setting of minimally invasive surgery. Furthermore, we found the model to be suitable for future training with the product before clinical application after product approval. Specifically, because other training models, such as simulation, have not advanced to the point in which the operator’s experience includes managing real-life pathophysiology. This animal model provides a unique learning experience and was effective to allow the operator to perform procedures on a variety lesion types with a variety of bleeding severity. The technical skills gained from this study can be used to support robotic liver surgery procedures in humans.

## Conclusions

The data from this preclinical study provide evidence that hemostasis of up to life-threatening bleeding during robotic liver surgery can be achieved quicker and more reliably with the NHS-POx-loaded patch when compared to the standard of care product, a fibrin sealant patch. In addition to the hemostatic performance, the lack of preparation time and the ease-of-use of the NHS-POx patch, even in minimally invasive settings, may help reduce the need for conversion to open surgery to control bleedings and improve clinical outcomes. Future studies are needed to evaluate the use of GATT-Patch in clinical procedures.

### Supplementary Information

**Additional file 1.**

## Data Availability

All data generated or analyzed during this study are included in this published article.
